# Supplementary material for: Practices of tablet splitting and dose uniformity of fragments at public hospitals in Ethiopia: A cross-sectional study supported by experimental findings
Source: PLoS One. 2022 Dec 15;17(12):e0279135. doi: 10.1371/journal.pone.0279135 (PMC9754160; doi:10.1371/journal.pone.0279135)
Supplement: S1 Data — (PDF) [file pone.0279135.s001.pdf]

S 1 table: Data on Intact and fragments (two halves) of furosemide 40mg tablets

No.	Tablets split by the patient				Tablets split by pharmacist			
	Weight of intact tablets	Weight of fragment 1 (F1)	Weight of fragment 2 (F2)	Weight loss upon splitting	Weight of intact tablets	Weight of fragment 1 (F1)	Weight of fragment 2 (F2)	Weight loss upon splitting
1	0.189	0.1	0.085	0.004	0.197	0.097	0.099	0.001
2	0.19	0.112	0.077	0.001	0.196	0.103	0.091	0.002
3	0.21	0.1	0.08	0.03	0.203	0.1	0.097	0.003
4	0.188	0.099	0.086	0.003	0.203	0.099	0.098	0.006
5	0.2	0.11	0.072	0.018	0.2	0.109	0.082	0.009
6	0.21	0.132	0.062	0.016	0.209	0.1	0.099	0.011
7	0.2	0.09	0.099	0.011	0.2	0.096	0.099	0.005
8	0.2	0.1	0.098	0.002	0.189	0.1	0.088	0.001
9	0.205	0.07	0.13	0.005	0.205	0.098	0.101	0.006
10	0.197	0.052	0.14	0.005	0.195	0.097	0.094	0.004
11	0.202	0.09	0.108	0.004	0.204	0.095	0.108	0.001
12	0.201	0.1	0.1	0.001	0.199	0.1	0.098	0.001
13	0.195	0.079	0.11	0.006	0.195	0.089	0.1	0.006
14	0.194	0.098	0.09	0.006	0.197	0.098	0.09	0.009
15	0.197	0.12	0.07	0.007	0.197	0.1	0.093	0.004
16	0.203	0.11	0.09	0.003	0.208	0.11	0.093	0.005
17	0.205	0.08	0.12	0.005	0.204	0.09	0.1	0.014
18	0.205	0.09	0.109	0.006	0.206	0.099	0.105	0.002
19	0.21	0.102	0.105	0.003	0.209	0.102	0.105	0.002
20	0.199	0.1	0.098	0.001	0.199	0.1	0.098	0.001
21	0.197	0.07	0.125	0.002	0.195	0.09	0.101	0.004
22	0.202	0.102	0.099	0.001	0.204	0.102	0.099	0.001
23	0.189	0.08	0.087	0.022	0.196	0.085	0.098	0.009
24	0.198	0.097	0.1	0.001	0.196	0.095	0.1	0.001
25	0.2	0.1	0.098	0.002	0.2	0.1	0.098	0.002
26	0.207	0.098	0.105	0.004	0.204	0.098	0.105	0.001
27	0.203	0.08	0.1	0.013	0.203	0.089	0.1	0.014
28	0.197	0.101	0.097	0.009	0.199	0.101	0.097	0.001
29	0.19	0.084	0.105	0.001	0.19	0.089	0.1	0.001
30	0.204	0.099	0.098	0.007	0.203	0.099	0.098	0.006
	Mean = 0.200 SD = 0.0063 %RSD = 3.15	Mean = 0.0964 SD = 0.0167 %RSD = 17.6		Mean = 0.0066	Mean = 0.200 SD = 0.0060 %RSD = 3.00	Mean = 0.098 SD = 0.0054 %RSD = 5.5		Mean = 0.0044_

S2 table: Data on Intact and fragments (two halves) of Haloperidol 5mg tablets

No.	Tablets split by the patient				Tablets split by pharmacist			
	Weight of intact tablets	Weight of fragment 1 (F1)	Weight of fragment 2 (F2)	Weight loss upon splitting	Weight of intact tablets	Weight of fragment 1 (F1)	Weight of fragment 2 (F2)	Weight loss upon splitting
1	0.1	0.052	0.045	0.003	0.099	0.05	0.048	0.001
2	0.099	0.061	0.037	0.001	0.099	0.057	0.041	0.001
3	0.098	0.045	0.05	0.005	0.103	0.048	0.05	0.005
4	0.104	0.049	0.048	0.007	0.104	0.049	0.046	0.009
5	0.101	0.041	0.052	0.008	0.1	0.042	0.052	0.006
6	0.101	0.055	0.042	0.004	0.098	0.05	0.045	0.003
7	0.103	0.05	0.049	0.004	0.101	0.055	0.043	0.001
8	0.096	0.047	0.047	0.002	0.1	0.046	0.048	0.006
9	0.097	0.05	0.042	0.005	0.097	0.05	0.045	0.002
10	0.1	0.042	0.055	0.003	0.1	0.051	0.047	0.002
11	0.102	0.049	0.048	0.005	0.101	0.049	0.048	0.004
12	0.098	0.05	0.046	0.002	0.099	0.05	0.047	0.002
13	0.095	0.049	0.04	0.006	0.098	0.049	0.046	0.003
14	0.097	0.048	0.049	0	0.098	0.048	0.049	0.001
15	0.097	0.053	0.04	0.004	0.097	0.05	0.042	0.005
16	0.103	0.038	0.06	0.005	0.1	0.048	0.048	0.004
17	0.105	0.054	0.05	0.001	0.105	0.05	0.05	0.005
18	0.105	0.05	0.049	0.006	0.103	0.05	0.049	0.004
19	0.095	0.042	0.045	0.008	0.096	0.042	0.045	0.007
20	0.099	0.049	0.048	0.002	0.095	0.047	0.047	0.001
21	0.097	0.05	0.045	0.002	0.097	0.05	0.045	0.002
22	0.1	0.042	0.056	0.002	0.1	0.047	0.051	0.002
23	0.101	0.05	0.047	0.004	0.101	0.05	0.048	0.003
24	0.098	0.056	0.04	0.002	0.099	0.057	0.04	0.002
25	0.099	0.049	0.047	0.003	0.097	0.056	0.04	0.001
26	0.102	0.048	0.045	0.009	0.102	0.048	0.045	0.009
27	0.104	0.054	0.043	0.007	0.1	0.05	0.045	0.005
28	0.099	0.041	0.056	0.002	0.105	0.042	0.062	0.001
29	0.1	0.044	0.053	0.003	0.1	0.045	0.051	0.004
30	0.103	0.041	0.058	0.004	0.1	0.05	0.048	0.002
	Mean = 0.100<br>SD = 0.0028<br>%RSD = 2.8	Mean = 0.048<br>SD = 0.0054<br>%RSD = 11.25		Mean = 0.0040	Mean = 0.100<br>SD = 0.0028<br>%RSD = 2.8	Mean = 0.048<br>SD = 0.0041<br>%RSD = 8.5		Mean = 0.0036
