# Supplementary material for: Practices of tablet splitting and dose uniformity of fragments at public hospitals in Ethiopia: A cross-sectional study supported by experimental findings
Source: PLoS One. 2022 Dec 15;17(12):e0279135. doi: 10.1371/journal.pone.0279135 (PMC9754160; doi:10.1371/journal.pone.0279135)
Supplement: Attachment — Submitted filename: Response to reviewers.docx [file pone.0279135.s002.docx]

Response to Reviewers

**Tablet dosage form splitting practices and dose uniformity of fragments at public hospitals in Ethiopia: a Mixed cross-sectional and experimental study methods**
The authors would like to thank reviewers for their insightful, constructive comments. In the preparation of this response, the Reviewer’s comments have been numbered as indicated in the appended annotated copies of the Reviewer’s evaluations and supporting files.

**Response to Editors**

We note that you have provided funding information that is not currently declared in your Funding Statement. However, funding information should not appear in the Acknowledgments section or other areas of your manuscript. We will only publish funding information present in the Funding Statement section of the online submission form. Please remove any funding-related text from the manuscript and let us know how you would like to update your Funding Statement. Currently, your Funding Statement reads as follows: “The author(s) received no specific funding for this work.”

The authors acknowledge the editor’s comment. The authors did not receive funding from Addis Ababa University. However, the university supports us by providing ethical clearance to undertake the study. We modified the acknowledge part accordingly.

Response to reviewer 1

The authors appreciated the reviewer’s comments

The study is well organized however the topic is vast more drugs can be selected for better comparison. The author can give the reason of choosing the specific drugs for the study.

The authors accepted the reviewer’s comment and modified the title accordingly. The authors select two drugs (Furosemide and haloperidol) because of their high potency, were commonly split based on the data obtained from the primary cross-sectional study and were available during the study period.

On what basis these two hospitals are selected?

Both hospitals were purposively selected for the study based on their high patient flow, their willingness to be enrolled in the study and the setup of pharmacy units were suitable and found near to access information easily.

Questionnaire distributed in pharmacy and hospitals does not include similar questions.

The aim of the **cross-sectional study section** is not to compare the knowledge of pharmacy professionals with patients. Hence, the contents of the questionnaires are different for patients and pharmacists.

Response to reviewer 2

The authors appreciated the reviewer’s comments

1. English need extensive revision.

Thank you, we revised accordingly.

2. Abstract and conclusion should be re-written according to results

Thank you, revised accordingly.

3. Method:
   A. Please enlist the studies which were considered to develop questionnaire?

The authors accepted the reviewer’s comment

B. What were the results of reliability testing and C) How the validity was monitored?

The authors accepted the reviewer’s comment. We included methods we followed to assure the reproducibility and validity of our test. “For validity and linearity of the method, 6 different concentrations (1μg/ml, 2μg/ml, 4μg/ml, 6μg/ml, 8μg/ml and 10μg/ml) were prepared by serial dilution from a standard working solution of the drug in 0.1 M sodium hydroxide for furosemide tablet.” The repeatability of the test was assured by 6 determinations at 100% of test concentration.

D. How the same questionnaire justifies the healthcare workers and patients?

The contents of the questionnaire were different for Pharmacists and patients.

E. How the participant were selected? And What were the rational to exclusion and inclusion criteria ?

Thank you for your comments. The authors modified the exclusion and inclusion criteria. The participants were selected with clearly justified inclusion and exclusion criteria for both cross-sectional and experimental studies.
